# Caution: Reptile pets shuttle grasshopper allergy and asthma into homes

**DOI:** 10.1186/s40413-015-0072-1

**Published:** 2015-08-17

**Authors:** Erika Jensen-Jarolim, Isabella Pali-Schöll, Sebastian A.F. Jensen, Bruno Robibaro, Tamar Kinaciyan

**Affiliations:** Comparative Medicine, Messerli Research Institute, University of Veterinary Medicine Vienna, Medical University Vienna and University Vienna, Vienna, Austria; Institute of Pathophysiology and Allergy Research, Center of Pathophysiology, Infectiology and Immunology, Medical University Vienna, Waehringer G. 18-20, 1090 Vienna, Austria; AllergyCare, Allergy Diagnosis and Study Center, 1220 Vienna, Austria; The Rudolfinerhaus, Vienna, Austria; Division of Immunology, Allergy and Infectious Diseases, Department of Dermatology, Medical University Vienna, Vienna, Austria

**Keywords:** Grasshopper, Reptile, Asthma, Pets, Reptile pets, IgE, Allergy

## Abstract

The numbers of reptiles in homes has at least doubled in the last decade in Europe and the USA. Reptile purchases are increasingly triggered by the attempt to avoid potentially allergenic fur pets like dogs and cats. Consequently, reptiles are today regarded as surrogate pets initiating a closer relationship with the owner than ever previously observed. Reptile pets are mostly fed with insects, especially grasshoppers and/or locusts, which are sources for aggressive airborne allergens, best known from occupational insect breeder allergies. Exposure in homes thus introduces a new form of domestic allergy to grasshoppers and related insects. Accordingly, an 8-year old boy developed severe bronchial hypersensitivity and asthma within 4 months after purchase of a bearded dragon. The reptile was held in the living room and regularly fed with living grasshoppers. In the absence of a serological allergy diagnosis test, an IgE immunoblot on grasshopper extract and prick-to-prick test confirmed specific sensitization to grasshoppers. After 4 years of allergen avoidance, a single respiratory exposure was sufficient to trigger a severe asthma attack again in the patient.

Based on literature review and the clinical example we conclude that reptile keeping is associated with introducing potent insect allergens into home environments. Patient interviews during diagnostic procedure should therefore by default include the question about reptile pets in homes.

## Review

### Reptile pets and their allergenic food

Insects as alien species may represent transient threats, or get invasive and established [1]. Trade and transport play an important role in their dispersal as contamination or as goods [2, 3]. Insects represent a growing market segment, not only because edible insects likely in the future will be increasingly popular for our own food, but also because insects have since long been used in industrial feeds for domestic animals such as poultry [4], or for reptiles in research labs and homes. In this context, the production of a single cricket farm raised from 10 million animals a week in 2004 up to 25 million in 2012, according to a report by the European Association of Reptile Keepers (EURAK) [5]. Pet stores and pet superstores distribute these insects, all while alive. Thus in parallel with the strong trend towards home reptiles in the US and Europe [6], these “alien” insect species have been introduced on purpose in our ultimate living environment as feed for reptiles. It may be doubted that through this rapid change any reasonable co-evolutionary interaction outcome may take place [7], but rather unwanted side effects could occur.

The number of insects actually living in our homes is not known, and can only be estimated indirectly by correlation to the number of domestic reptiles. The report of the White House quotes the American Pet Products Association, according to which “pet reptile” owning US households increased by 68 %, from 2.8 million households in 1994 to 4.7 million in 2008 [6]. According to EURAK, 8 million reptile pets are kept in UK in 1.1 million households [5]. Reptiles have also become popular in Europe. In a statement on *Salmonella* infections via exotic reptiles, the Robert Koch-Institute quotes the German Industry association of Pet market, saying that purchases of terraria have increased by 6.1 % in 2008, that 1.2 % of German households own reptiles, and that especially keeping of dragon species (genus *Pogona*, family *Agamidae*), or *Iguanidae* has increased tenfold [8].

Considering that on average 4 living grasshoppers with each a weight of 0.5 g are fed to a medium size dragon per week, 1.120 kg grasshoppers are eaten by a single reptile during its life time of optimally 10 years.

Reptile holders were classified as i) beginners who intend to buy an easy-to-handle and non-expensive animal, ii) enthusiasts with deep interest and knowledge in reptiles, and iii) hobbyists who also engage in breeding [6]. We may here add another category, namely iv) families with pet allergies or increasing awareness of allergies to furry animals [9]. The acquisition of a reptile may then be associated with a change of the attitude towards reptiles from biological interest to strong emotional binding to the surrogate pet [10, 6]. The close contact between child, owner and reptile results in contamination of textiles and furniture with leftovers of the insect feed from skin and feces of the reptile. This is not only a problem in terms of *Salmonella* infection [8], but grasshoppers have since long also been known to pose an allergenic risk: The first observation of grasshopper allergy was reported in 1953 [11], shortly after followed by the understanding that these insects may cause allergic asthma in occupational settings [12]. For instance locusts, grasshoppers and cricketts are bred for research laboratories [13, 14] or for reptile feed market [6, 5]. It was perceived that especially a high respiratory exposure with dust of insects including grasshoppers is associated with a risk for allergic rhinoconjunctivitis and bronchial hyperreactivity and asthma [15, 16]. In this study, sensitization was seen in 43.8 % of occupationally exposed workers as compared to 3.8 % of control subjects. Further the skin test correlated better with asthma symptoms than cutaneous symptoms in 4 workers with asthma. Whereas contact allergy is mainly due to an underlying type IV reactivity and may result from occupational exposure [17], the present review focuses on immediate type (type I) allergies.

Of 15 occupationally exposed grasshopper workers, contacts with the allergen provoked acute respiratory and cutaneous symptoms in five of them [18]. The IgE-binding allergens were described in terms of molecular mass, but not further identified. Interestingly, the authors demonstrated that air-sampler filters in the grasshopper breeding room could capture allergen, and that it corresponds to antigens derived from locust gut.

The question whether natural migration of locust swarms may produce allergen levels that even result in asthma [19] is hotly debated. In their own study, 6 of 10 laboratory workers exposed to African grasshopper *Locusta migratoria* showed symptoms ranging from urticaria to rhinoconjunctivitis. It could be demonstrated that locust allergens (and particularly a newly described 70 kDa allergen) were especially contained in the wings of the animals, but also in the feces.

In a British study, 32 workers of a food supplier, breeding insects for exotic pets, were investigated [20], with 34 % reporting work-related symptoms. The dust levels reached 1.2–17.9 mg/m^3^ with concomitantly high endotoxin levels of up to 29.43 kEU/m^3^ [21], compared to the endotoxin concentration of 18.0 kEU/m^3^ in an average classroom [22]. This is interesting as principally, via toll-like receptor 4 (TLR4) the release of TSLP (thymic stromal lymphopoietin) from bronchial epithelia could be triggered supporting Th2 skewing [23]. Similarly, grasshopper allergens especially in context with endotoxin could lead to respiratory hypersensitivity.

Grasshopper allergy has been discussed also in the pediatric population [24, 25]. When asthmatic US children were screened by RAST discs coupled with insect extracts for specific IgE, 7 of 36 (19 %) asthmatics reacted to grasshopper extracts, however, without approval of a clinical relevance [26]. In 2009 Prasad et al. skin-pricked 2880 Indian patients with allergic rhinoconjunctivitis and revealed that insects were the most important cause of sensitization in 21.1 % of tested patients, with 20.8 % of them being specifically sensitized against grasshoppers [27].

When sera from patients with a history of respiratory allergies to insects of several kinds (house fly, blowfly, clothes moth, warehouse moth, cockroach, carpet beetle, silverfish) were tested for IgE binding, 30 % reacted with all seven species, and 50 % reacted with four extracts. Comparison with additional 11 species including Australian plague locust *(Chortoicetes terminifera)* revealed that common crossreactive allergens are present in different species possibly responsible for a “pan allergy” to insects [28]. This concept was supported by the identification of the pan allergen tropomyosin in the study of Leung, when nine shrimp allergic sera reacted also to insects including long-horned grasshopper (*Tettigoniidae*), cockroach and fruit fly [29]. Pener et al. in their comprehensive review on allergy to locusts and acridid grasshoppers expressed the urgent need that “the molecular structure of the allergens” should be revealed [30].

### Clinical example: Specific sensitization to grasshopper in a reptile home

**Fig. 1:**
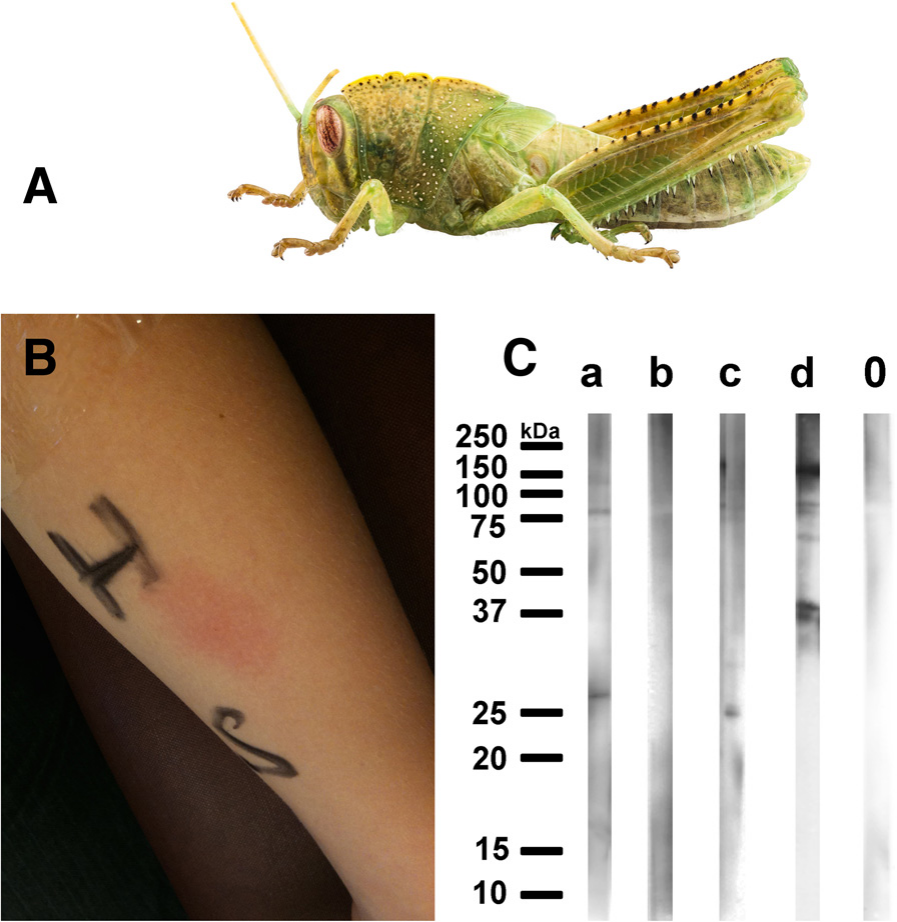
**a** Nymph of Egyptian locust species *Anacridium aegyptium* (Fotolia© paulrommer). **b** Confirmation of specific sensitization by Prick-to-Prick test with Egyptian grasshopper.Crushed wing material of a frozen Egyptian locust (H) and saliva of the boy’s bearded dragon Sony (S) was pricked on the forearm of the patient, in addition to the standard Prick test with inhalant allergens. **c** Reactivity of patient’s IgE on blotted extracts of migratory grasshopper (*Locusta migratoria*) legs (a) and wings (b), and from Egyptian locust (*Anacridium aegypticum)* legs (c) and wings (d). As negative control, a serum of a non-allergic person was tested (0). Bound IgE was detected by peroxidase-labeled anti-IgE antibody and the reaction developed with ECL

A bearded dragon was purchased by the parents of an eight-year old boy (family A) as pet surrogate and installed with its terrarium in the living room of an apartment in Vienna. Four months later, the boy experienced several episodes during the nights awaking with glottal edema and wheezing, prompting the parents to refer to a children’s hospital in Vienna, Austria, where the situation could be controlled with beta-2-mimetic aerosol and rectal hydrocortisone. The provisory diagnosis was “pseudo croup presumably elicited by viral infections”. We carefully elaborated the case investigating all possible respiratory allergen sources including pets. The only pet was bearded dragon “Sony”, being fed with 3–4 living grasshoppers a week. We performed a Prick test with standard inhalant allergen series and Prick-to-Prick test using the wing of one Egyptian grasshopper, the species being fed the reptile by the boy, and the saliva of the bearded dragon. Only Prick-to-Prick test with the wing of the grasshopper elicited a pronounced wheal and flare reaction (Fig. 1a), all other skin tests resulted negative. The boy was atopic (total IgE level: 168 kU/l); CAP RAST FEIA: rye and grass pollen class II, birch and mugwort pollen class I without any clinical relevance; on an ISAC ImmunoCAP microarray minimal sensitization to grass and cypress pollen again without clinical relevance could be detected, but no IgE-reactivity to any of the insect or mollusk tropomyosins. As no commercial IgE diagnostics for grasshopper allergy was available we extracted proteins in PBS containing protease inhibitors EDTA and EACA from either wings or legs from Egyptian grasshoppers *(Anacridium aegyptium, AA)* or from migratory locust (*Locusta migratoria, LM*). Extracts were lyophilized and freeze dried until use, separated at 50 microg/lane on a 15 % reducing SDS-PAGE and blotted to nitrocellulose. Fig. 1b shows that patient’s IgE was directed against 27, 80 and 125 kDa proteins of LM leg extract (lane A), and to proteins > 250 kDa in LM wing extract (B); in AA leg extract IgE detected bands at 25, 75 and 90 kDa (C), and in AA wing extract at a molecular weight of 36, 38, 70, 76, 90, and 152 kDa (D). We did not detect IgE binding to recombinant shrimp tropomyosin or troponin in the immunoblot (data not shown).

The patient’s parents were encouraged to get rid of the animal due to the severity of the allergic reaction in the absence of any causal treatment. The bearded dragon was therefore transferred to family B in the same house, and the allergic boy did never since enter their apartment. However, 4 years after the first event, the boy (now aged 12) stopped by at the door of family B, who still owned the bearded dragon plus several snakes and other reptiles. Inhalation of warm air streaming out of this apartment to the colder corridor resulted in shortened breath due to acute bronchial obstruction: approx. 4 h later the patient awoke with wheezing being recognized and treated by the parents with 100 mg intra-rectal hydrocortisone. The following day, when the boy’s respiratory condition was stable and the lung function almost recovered, the persisting grasshopper sensitization was confirmed in a skin prick test.

The patient was released with a prophylactic prescription of beta-2-mimetics aerosol, oral anti-histamines, rectal hydrocortisone and an epinephrin autoinjector.

## Conclusion

There is strong evidence that grasshoppers have a high allergenic potential and increasingly are invading our homes together with the reptile pets. So far, besides occupational grasshopper allergies no private sensitizations were described, but have to be expected considering the great market growth of reptiles and associated insects for feed. We evidence here that indeed via grasshoppers as feed for reptiles within a short time period a highly specific, clinically relevant hypersensitivity with severe asthma can be induced with a long-term memory. The reptile keeping in the home results in airborne dissemination of allergens from wings, legs and -according to the literature [30] - from the peritrophic envelope of feces, and at an allergen level sufficient for sensitization as well as triggering of symptoms.

### Consent

Written informed consent was obtained from the patient and his parents for this publication and accompanying images. A copy of the written consent is available for review by the Editor-in-Chief of this journal.

## Abbreviations

AA: Anacridium aegyptium
EACA: Epsilon-aminocaproic acid (EACA)
ECL: Enhanced chemiluminescence
EDTA: Ethylenediamine tetraacetic acid
EURAK: European Association of Reptile Keepers
LM: Locusta migratoria
PBS: phosphate buffered saline
RAST: Radioallergosorbent test
SDS-PAGE: Sodium dodecyl sulfate-polyacrylamide electrophoresis
TLR: Toll-like receptor
TSLP: Thymic stromal lymphopoietin
US: United States
